# VAMP8 suppresses the metastasis via DDX5/β-catenin signal pathway in osteosarcoma

**DOI:** 10.1080/15384047.2023.2230641

**Published:** 2023-07-05

**Authors:** Shuo Yang, Ping Zhou, Lelei Zhang, Xiangpeng Xie, Yuanyi Zhang, Kaida Bo, Jing Xue, Wei Zhang, Faxue Liao, Pengfei Xu, Yong Hu, Ruyu Yan, Dan Liu, Jun Chang, Kecheng Zhou

**Affiliations:** a Department of Orthopaedics, The First Affiliated Hospital of Anhui Medical University, Hefei, China; b Department of Orthopaedics, Anhui Public Health Clinical Center, Hefei, China; c Clinical Pathology Center, The First Affiliated Hospital of Anhui Medical University, Hefei, China; d School of Basic Medical Sciences, Anhui Medical University, Hefei, China; e Cancer Metabolism Laboratory, School of Life Sciences, Anhui Medical University, Hefei, China

**Keywords:** Osteosarcoma, VAMP8, metastasis, DDX5, β-catenin

## Abstract

Osteosarcoma is a highly metastatic malignant bone tumor, necessitating the development of new treatments to target its metastasis. Recent studies have revealed the significance of VAMP8 in regulating various signaling pathways in various types of cancer. However, the specific functional role of VAMP8 in osteosarcoma progression remains unclear. In this study, we observed a significant downregulation of VAMP8 in osteosarcoma cells and tissues. Low levels of VAMP8 in osteosarcoma tissues were associated with patients’ poor prognosis. VAMP8 inhibited the migration and invasion capability of osteosarcoma cells. Mechanically, we identified DDX5 as a novel interacting partner of VAMP8, and the conjunction of VAMP8 and DDX5 promoted the degradation of DDX5 via the ubiquitin-proteasome system. Moreover, reduced levels of DDX5 led to the downregulation of β-catenin, thereby suppressing the epithelial–mesenchymal transition (EMT). Additionally, VAMP8 promoted autophagy flux, which may contribute to the suppression of osteosarcoma metastasis. In conclusion, our study anticipated that VAMP8 inhibits osteosarcoma metastasis by promoting the proteasomal degradation of DDX5, consequently inhibiting WNT/β-catenin signaling and EMT. Dysregulation of autophagy by VAMP8 is also implicated as a potential mechanism. These findings provide new insights into the biological nature driving osteosarcoma metastasis and highlight the modulation of VAMP8 as a potential therapeutic strategy for targeting osteosarcoma metastasis.

## Introduction

Osteosarcoma (OS) is a highly malignant bone tumor and frequently occurs in children and adolescents with a high rate of recurrence and metastasis.^1^ OS originates from primitive mesenchymal cells originating in bone, and in rare cases from soft tissue.^2^ The incidence of OS is estimated at 2–4 cases per million per year, with a peak occurrence between the ages of 15 and 19 y.^3^ The introduction of systemic chemotherapy has improved the 5-y survival rate for OS to 60%–70%.^3^ However, the 5-y survival rate remains low at 20% in metastatic OS patients.^4^ Tumor metastasis involves a complex process of dissemination from the primary tumor, transit within the circulatory system, colonization and establishment of metastasis.^5^ Despite advancements, the molecular mechanisms underlying OS metastasis remain poorly understood.

Soluble N-ethylmaleimide-sensitive factor activating protein receptors (SNAREs) classified into two groups: v-SNAREs (vesicle-SNARE) and t-SNAREs (target-SNARE). In yeast, there are 25 members of SNAREs, while in humans there are 36 members.^6,7^ SNAREs proteins primarily regulate the docking of the particles and vesicles to target membranes, as well as membrane fusion, which is essential for various biological processes, including viral infection, cell fertilization, intracellular transport and neurotransmitter release.^8^ Vesicle associated membrane protein 8 (VAMP8) is a v-SNAREs involved in the membrane fusion steps of various endosomes and secretions.^9,10^ Previous studies suggested that VAMP8 acts key roles in regulating cancer progression. For instance, VAMP8 inhibited lung cancer metastasis through the RAB37-mediated exocytosis of anti-metastasis cargos. Conversely, VAMP8 promoted tumor proliferation and temozolomide resistance by mediating autophagy in human glioma.^11,12^ These studies highlight the involvement of VAMP8 in cancer progression with essential functions. However, the roles of VAMP8 and its related molecular mechanism in OS remain unknown.

Asp-Glu-Ala-Asp (DEAD) box helicase 5 (DDX5) belongs to the DEAD-box protein family, which comprises vital RNA helicases.^13^ Previous studies have demonstrated that DDX5 facilitated cancer metastasis. Yang *et al*. reported that DDX5 activated β-catenin and promoted its nuclear translocation, subsequently inducing epithelial-mesenchymal transition (EMT)^14^. Moreover, DDX5 promoted tumor metastasis by reorganizing actin cytoskeleton and stimulating WNT/β-catenin signaling.^15–17^ Interestingly, elevated expression of DDX5 has been observed in OS patients and is associated with poor prognosis. Functional assays have showed that DDX5 promotes cell proliferation, migration and invasion and inhibits apoptosis in OS cells.^18–20^ However, it is unclear whether VAMP8 protein is involved in the function and regulation of DDX5.

Autophagy is a conserved catabolic pathway in which selected cellular components are degraded and recycled within lysosomes to maintain cellular homeostasis, with key regulatory functions during OS metastasis.^21–25^ However, the exact mechanisms of autophagy in OS metastasis remain incompletely understood *per se* and require further investigation.

In this study, we first analyzed the expression of VAMP8 in OS and found the downregulation of VAMP8. Through cellular experiments, we demonstrated that overexpression of VAMP8 significantly inhibited the capability of wound healing, migration and invasion. We further identified DDX5 as a major interacting partner of VAMP8 using Mass-Spectrometry and immunoprecipitation. Utilizing cycloheximide (CHX) assays and pharmacological approaches, we established that VAMP8 promotes the degradation of DDX5 via the ubiquitin-proteasome system. The degradation of DDX5 subsequently reduces the level of β-catenin and suppresses the EMT process. Additionally, our results indicate that VAMP8 enhances the autophagy process, which partly contributes to the metastasis-suppressive role. Taken together, our findings unraveled a novel tumor suppressor function of VAMP8 in OS and provide insights into potential molecular mechanisms involved.

## Results

### Downregulation of VAMP8 associates with the poor survival in OS patients

**Figure 1.**
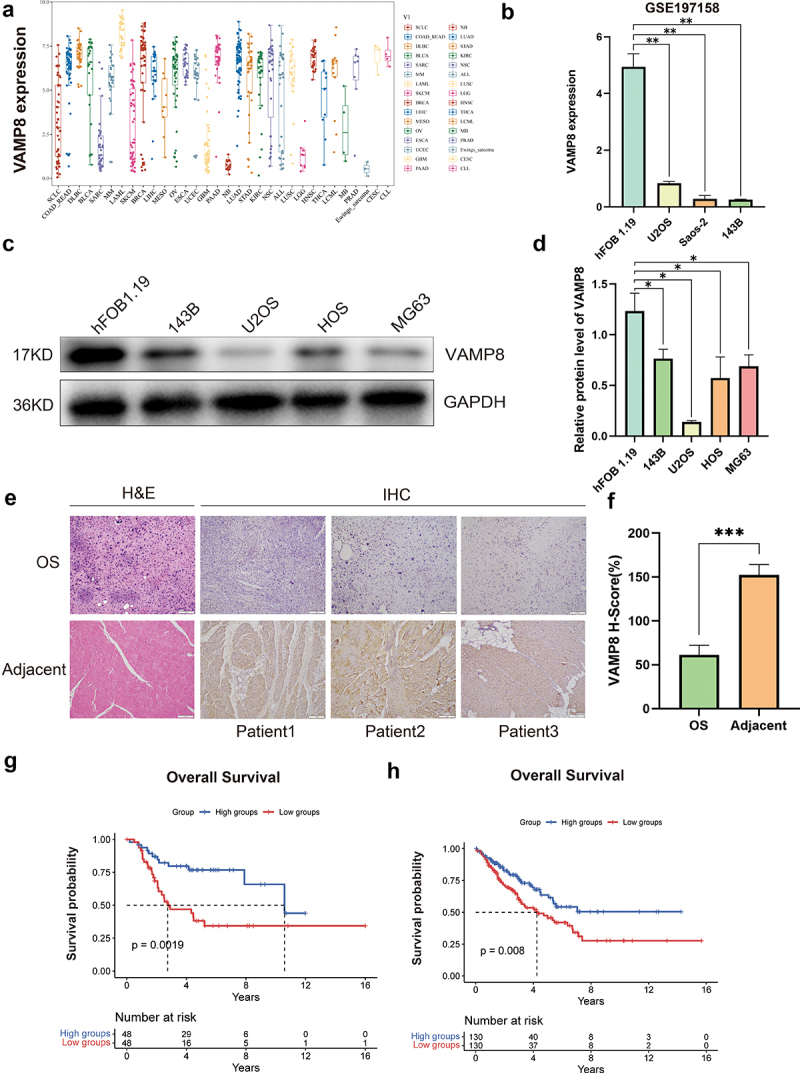
Downregulation of VAMP8 associates with the poor survival probability in OS.

To investigate the function of VAMP8 in OS, we initially analyzed the transcript levels of VAMP8 in Cancer Cell Line Encyclopedia (CCLE) database. The analysis revealed a downregulation of VAMP8 mRNA in sarcoma cells (Figure 1a). Consistently, data from Gene Expression Omnibus (GEO) database (GSE197158) demonstrated a significant reduction in VAMP8 mRNA expression in OS cells compared to the normal human osteoblast cell line hFOB1.19 (Figure 1b). Furthermore, immunoblot confirmed the downregulation of VAMP8 in OS cells (Figure 1c,d). Given the substantial decrease in expression observed in OS, we investigated whether VAMP8 plays a critical role in OS progression and patients’ survival. Therefore, we evaluated the protein level of VAMP8 in surgically resected OS tissues and adjacent normal tissues using immunohistochemistry. Our data revealed the decreased expression of VAMP8 in OS tissues, compared with the normal control tissues (Figure 1e,f). Additionally, we analyzed the clinical relevance regarding the abnormal expression of VAMP8 from Therapeutically Applicable Research To Generate Effective Treatments (TARGET) database. Interestingly, the low expression of VAMP8 in OS patients was significantly associated with poor overall survival rate (Figure 1g). This clinical relevance was also observed in sarcoma (Figure 1h). Collectively, these results indicated that VAMP8 might be involved in the progression of OS.

(a) The VAMP8 transcript levels in different types of cancer cells were obtained from the CCLE database.

(b) The VAMP8 mRNA levels in OS cells compared with hFOB1.19. Data were obtained from the GEO database (GSE197158), Mean ± SEM, p(hFOB1.19, U2OS) = 0.005, p(hFOB1.19, Saos-2) = 0.004, p(hFOB1.19, 143B) = 0.004.

(c-d) VAMP8 protein levels in hFOB1.19 and OS cells (143B, U2OS, HOS, MG63) were measured by Western blotting. Quantification of three experiments, Mean ± SEM, p(hFOB1.19, 143B) = 0.019, p(hFOB1.19, U2OS) = 0.011, p(hFOB1.19, HOS) = 0.016, p(hFOB1.19, MG63) = 0.028.

(e-f) VAMP8 protein levels in OS tissues and adjacent normal tissues were examined by H&E and IHC. Representative images were shown (magnification at × 40). Quantification of VAMP8 protein levels in paired clinical specimens, Mean ± SEM, *p* = .0002. Scale bar: 1 mm.

(g) Kaplan – Meier curves show the overall survival of patients with OS in terms of VAMP8 expression. Data were obtained from the TARGET database, *p* = .0019.

(h) Kaplan – Meier curves show the overall survival of patients with sarcoma in terms of VAMP8 expression. Data were obtained from the TCGA database, *p* = .008.

### Overexpression of VAMP8 inhibits OS cells migration and invasion

**Figure 2.**
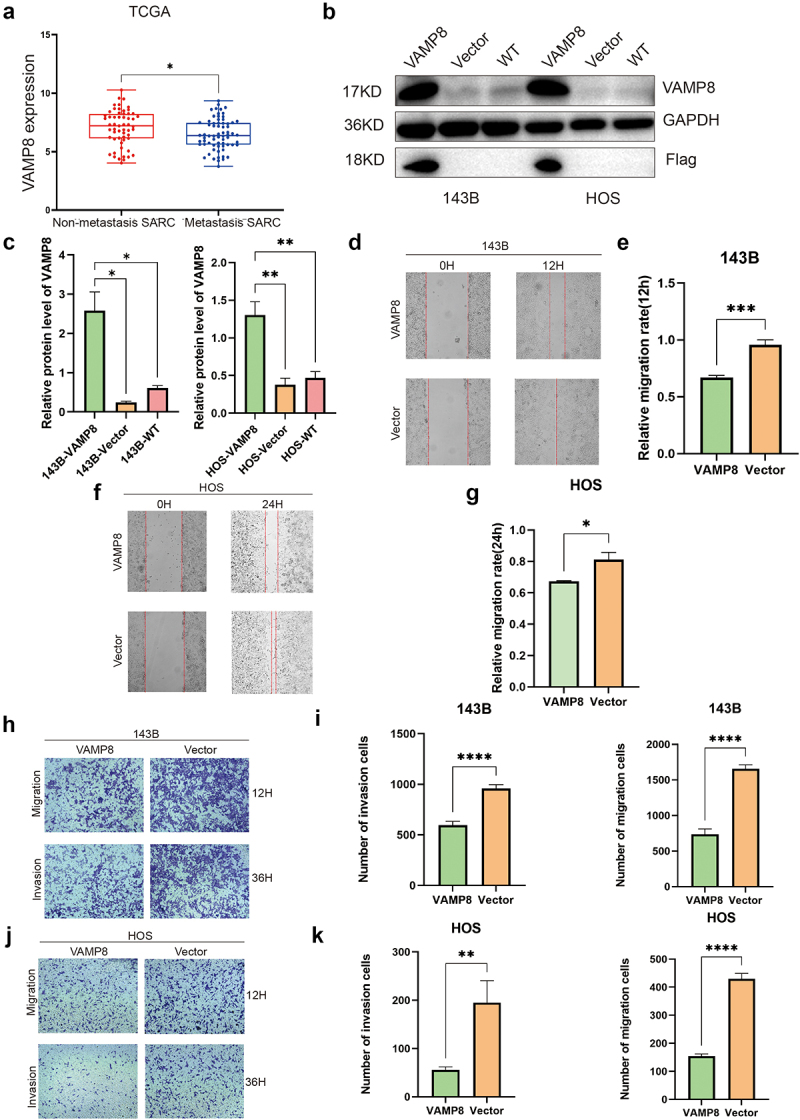
Overexpression of VAMP8 inhibits OS cells migration and invasion.

Given the significant downregulation of VAMP8 in OS and its clinical relevance, we investigated whether VAMP8 plays a role in regulating the cancer progression. To this end, we initially mined the public database (TARGET) and analyzed the expression of VAMP8 in metastatic OS and primary OS. The data reported VAMP8 mRNA tended to be lower in metastatic OS compared with primary OS; however, the difference was not statistically significant (Supplemental Fig S1). It is plausible the patients’ number in “metastatic OS” group is too limited to draw a reliable conclusion. We thus instead examined the VAMP8 expression from sarcoma cohorts in The Cancer Genome Atlas (TCGA) database and observed the significant downregulation of VAMP8 in metastatic sarcoma (Figure 2a).

(a) The VAMP8 mRNA levels in non-metastatic sarcoma and metastatic sarcoma tissues. Data were achieved from the TCGA database, Mean ± SEM, *p* = .03.

(b-c) The overexpression efficiency of VAMP8 in 143B and HOS cells was measured by Western blotting. The Flag tagged VAMP8 were confirmed and determined by Western blotting. Quantification of three experiments, Mean ± SEM, p(143B-VAMP8, 143B-Vector) = 0.018, p(143B-VAMP8, 143B-WT) = 0.021, p(HOS-VAMP8, HOS-Vector) = 0.009, p(HOS-VAMP8, HOS-WT) = 0.006.

(d-g) Wound healing assay was employed to measure the migratory capability of 143B and HOS cells. Representative images were shown (magnification at × 40). Quantification of three experiments, mean ± SEM, p(143B-VAMP8, 143B-Vector) = 0.0008, p(HOS-VAMP8, HOS-Vector) = 0.04.

(h-k) Transwell migration and matrigel invasion assays were used to determine the migratory and invasive ability of 143B and HOS cells. Representative images were shown (magnification at × 40). Quantification of three experiments, mean ± SEM, migration: p(143B-VAMP8, 143B-Vector)<0.0001, p(HOS-VAMP8, HOS-Vector)<0.0001; invasion: p(143B-VAMP8, 143B-Vector)<0.0001, p(HOS-VAMP8, HOS-Vector) = 0.008.

To further investigate the function of VAMP8, we generated VAMP8 stable cell lines overexpressing VAMP8 using a lentivirus system, and immunoblot experiment confirmed successful overexpression of Flag-tagged VAMP8 with 4–6 times higher protein levels (Figure 2b,c). Subsequently, we performed a series of experiments to study the role of VAMP8 in cancer cell migration and invasion. Wound healing experiments showed that wound closure rate was significantly reduced by VAMP8 overexpression in both 143B and HOS (Figure 2d-g). Furthermore, transwell assays with the addition of matrigel to mimic the in vivo extracellular environment demonstrated that overexpression of VAMP8 significantly reduced the invasive capability of 143B cells (Figure 2h,i). Similar results were observed in HOS cells (Figure 2j,k), indicating that the effect is not specific to a particular cell line. Collectively, our results indicate that VAMP8 inhibits the migratory and invasive capabilities of osteosarcoma cells.

### VAMP8 promotes DDX5 degradation via the ubiquitin-proteasome system

**Figure 3.**
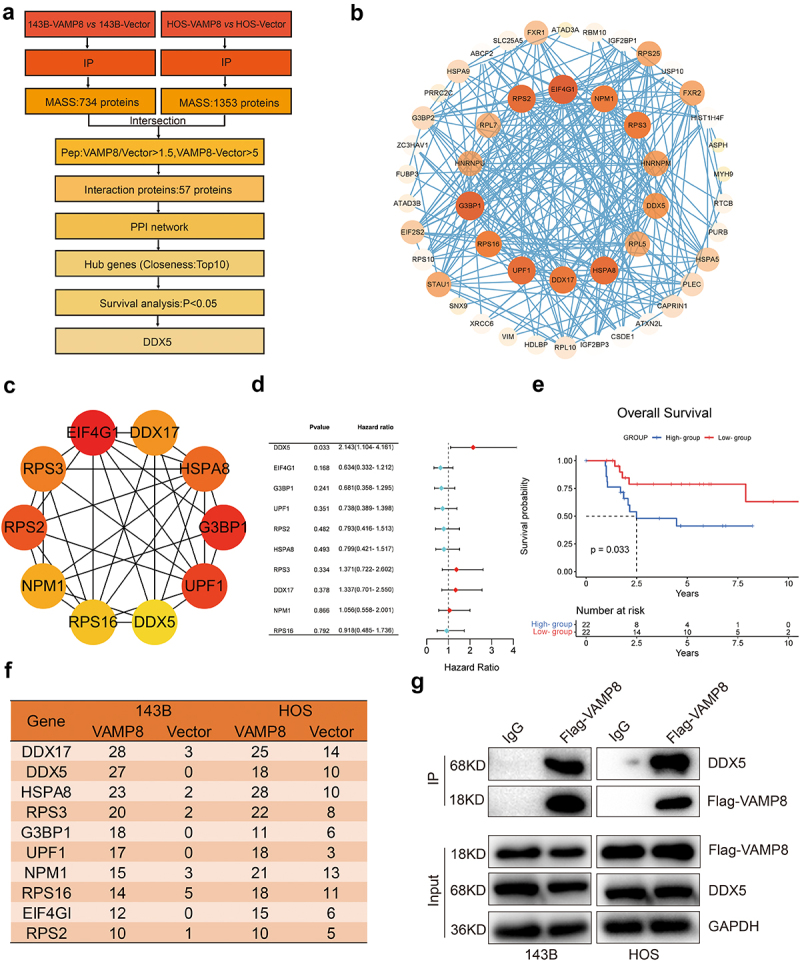
DDX5 was identified as an interacting partner of VAMP8.

To gain the mechanical insight into the VAMP8 suppressive functions of OS cells migration and invasion, we hypothesized that VAMP8 may interact with essential proteins involved in cell motion. To this end, immunoprecipitation experiments were performed using anti-Flag antibody to enrich VAMP8 interacting partners from 143B and HOS cells, followed by mass-spectrometry analysis to identify potential proteins (Figure 3a). Identified proteins were considered hits if the peptide count in VAMP8 expressing cells was at least 1.5-fold higher than the control group, with a minimum difference of five peptides (Figure 3a). A total of 57 potential interacting proteins of VAMP8 were identified.

(a) The VAMP8 mRNA levels in non-metastatic sarcoma and metastatic sarcoma tissues. Data were achieved from the TCGA database, Mean ± SEM, *p* = .03.

(b-c) The overexpression efficiency of VAMP8 in 143B and HOS cells was measured by Western blotting. The Flag tagged VAMP8 were confirmed and determined by Western blotting. Quantification of three experiments, Mean ± SEM, p(143B-VAMP8, 143B-Vector) = 0.018, p(143B-VAMP8, 143B-WT) = 0.021, p(HOS-VAMP8, HOS-Vector) = 0.009, p(HOS-VAMP8, HOS-WT) = 0.006.

(d-g) Wound healing assay was employed to measure the migratory capability of 143B and HOS cells. Representative images were shown (magnification at × 40). Quantification of three experiments, mean ± SEM, p(143B-VAMP8, 143B-Vector) = 0.0008, p(HOS-VAMP8, HOS-Vector) = 0.04.

(h-k) Transwell migration and matrigel invasion assays were used to determine the migratory and invasive ability of 143B and HOS cells. Representative images were shown (magnification at × 40). Quantification of three experiments, mean ± SEM, migration: p(143B-VAMP8, 143B-Vector)<0.0001, p(HOS-VAMP8, HOS-Vector)<0.0001; invasion: p(143B-VAMP8, 143B-Vector)<0.0001, p(HOS-VAMP8, HOS-Vector) = 0.008.

To explore the protein–protein interaction (PPI) among these 57 proteins, a PPI network was generated using the STING database and visualized with Cytoscape 3.9.0 (Figure 3b). The top 10 proteins with the highest closeness scores in the network were selected as hub genes (Figure 3c). We then conducted survival analysis on hub gene. Interestingly, DDX5 was the only significant protein among the hub genes (Figure 3d,e). Therefore, DDX5 was considered as a potential candidate in the further investigation. The detailed peptide number of DDX5 was higher in VAMP8 stably expressing cells compared with the control cells (Figure 3f). Moreover, immunoprecipitation experiment confirmed that VAMP8 indeed interacts with DDX5 (Figure 3g).

**Figure 4.**
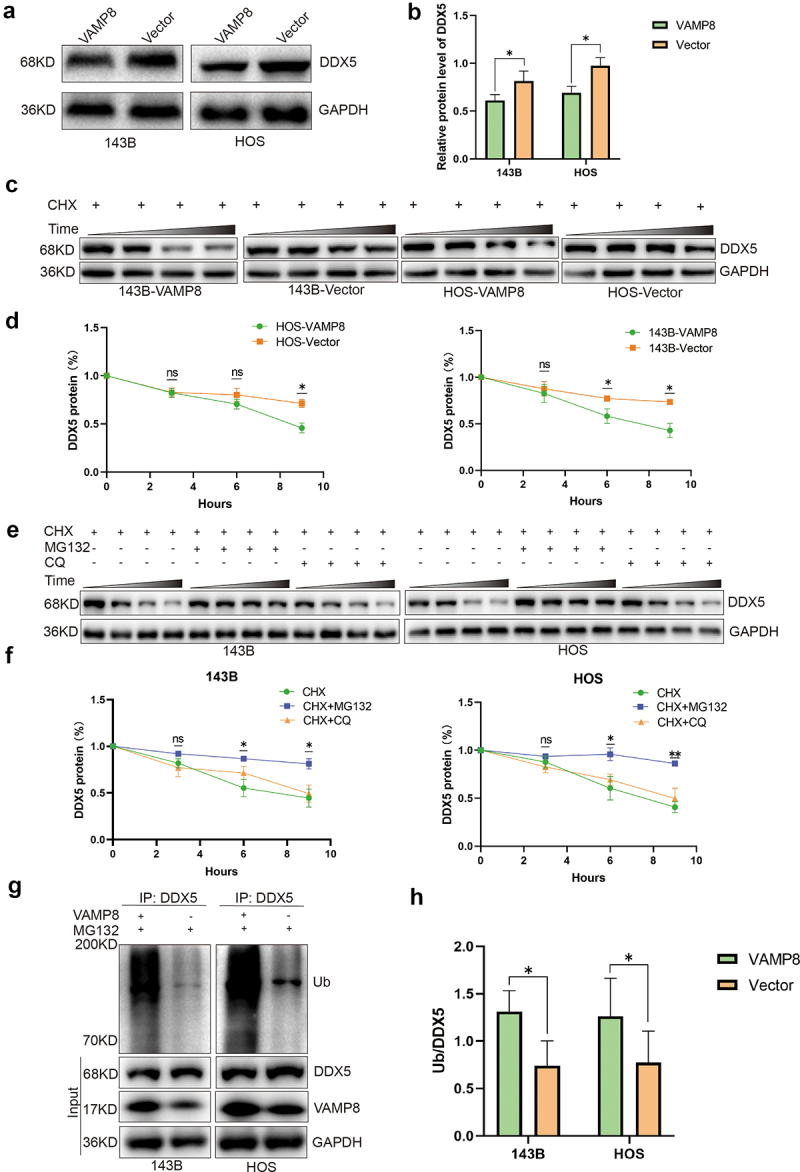
VAMP8 promotes DDX5 degradation via the ubiquitin-proteasome system.

We next examined whether the physical interaction between VAMP8 and DDX5 affected protein levels. Overexpression of VAMP8 significantly downregulated DDX5 protein levels in both 143B and HOS cells (Figure 4a,b). This suggested that VAMP8 may regulate DDX5 protein stability and degradation. To assess the protein half-life, we performed a cycloheximide (CHX) chase assay and found that VAMP8 expression reduced the half-life of DDX5 protein (Figure 4c,d). In order to further explore the pathway of DDX5 protein degradation, we treated cell with MG132 (proteasomal inhibitor, 10 μM) or chloroquine (CQ, lysosomal inhibitor, 25 μM), together with CHX. Our results showed that MG132 treatment, but not CQ treatment, significantly slowed down the degradation of DDX5, indicating that DDX5 was degraded via the ubiquitin-proteasome system (Figure 4e,f). We next measured the poly-ubiquitination level of DDX5. Cells were seeded and treated with MG132 to enhance the poly-ubiquitination, endogenous DDX5 was further enriched by immunoprecipitation and followed by Western blotting measuring ubiquitin level. Our experiments reported enhanced ubiquitin signals in VAMP8 stably expressing cells, indicating VAMP8 accelerates the poly-ubiquitination of DDX5 in OS cells (Figure 4g,h). Collectively, our data showed that VAMP8 directly interacts with DDX5 and promoted DDX5 degradation via the ubiquitin-proteasome system.

(a-b) Western blotting showed that overexpression of VAMP8 decreased DDX5 levels. Quantification of three experiments, Mean ± SEM, p(143B-VAMP8, 143B-Vector) = 0.021, p(HOS-VAMP8, HOS-Vector) = 0.017.

(c-d) Overexpression of VAMP8 cells were treated with 50 μg/ml CHX for 0, 3, 6 or 9 h. DDX5 levels were analyzed by western blotting. After treatment with CHX for 6 or 9 h, DDX5 levels was significantly reduced in 143B cells, and *p* values were 0.044 and 0.032, respectively. After treatment with CHX for 9 h, DDX5 levels was significantly reduced in HOS cells, and *p* value was 0.041. Quantification of three experiments, Mean ± SEM, “ns”: not significant.

(e-f) Western blotting analysis of DDX5 levels in VAMP8 stably expressing cells treated with CHX (50 μg/ml) with or without MG132 (10 μM) and CQ (25 μM) for 0, 3, 6 or 9 h. MG132 treatment for 6 or 9 h, but not CQ treatment, significantly slowed down the degradation of DDX5, and *p* values were 0.04 and 0.032 in 143B cells, 0.013 and 0.007 in HOS cells, respectively. Quantification of three experiments, Mean ± SEM, “ns”: not significant.

(g-h) After treatment with MG132 (10 μM) for 9 h, 143B and HOS lysates were subjected to immunoprecipitation (IP) with anti-DDX5 antibody. Ubiquitin, DDX5, VAMP8 and GAPDH levels were analyzed by immunoblot. Quantification of three experiments, Mean ± SEM, p(143B-VAMP8, 143B-Vector) = 0.027, p(HOS-VAMP8, HOS-Vector) = 0.03.

### VAMP8 regulates osteosarcoma cells migration and invasion through DDX5/β-catenin pathway and the inhibition of EMT

**Figure 5.**
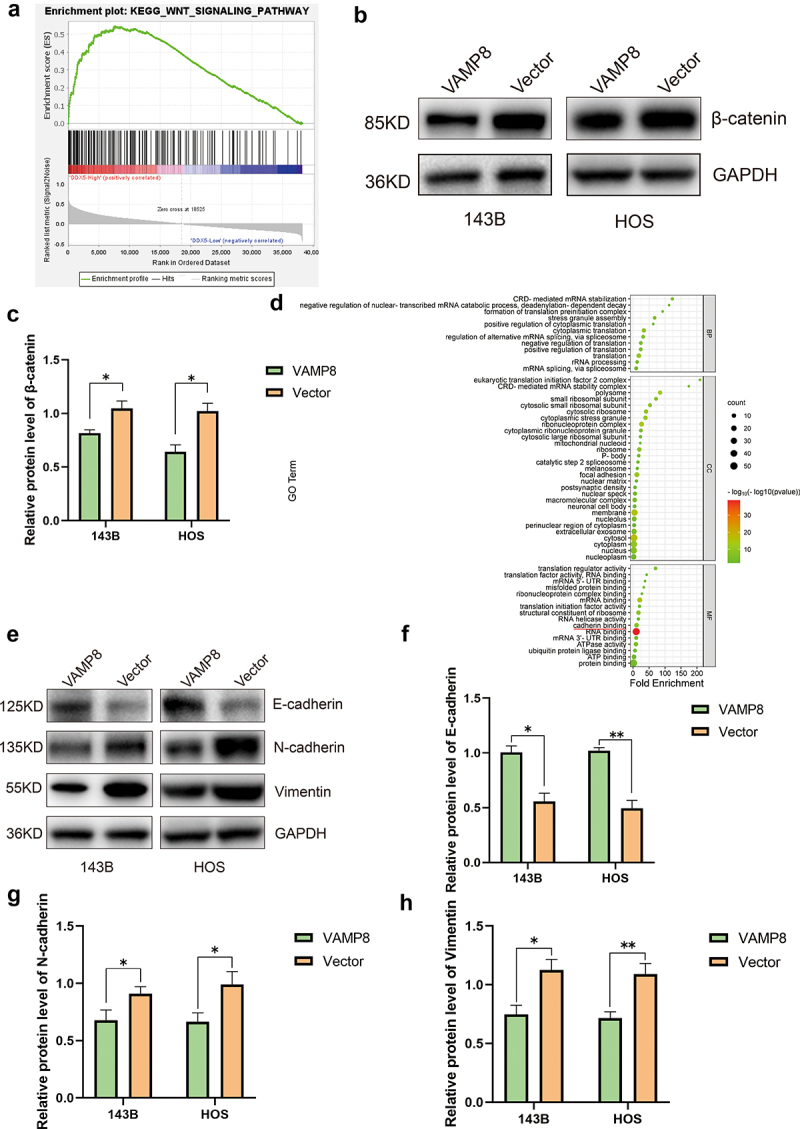
VAMP8 regulates osteosarcoma cells migration and invasion through DDX5/β-catenin pathway and the inhibition of EMT.

To elucidate the detailed mechanisms underlying VAMP8‘s role in OS metastasis and invasion, we conducted gene set enrichment analysis (GSEA) using data from the TARGET database. The results revealed that low expression of VAMP8 was enriched in the WNT pathway, although the *P* value did not reach significance (Supplemental Fig S2). Interestingly, the high expression of DDX5 was significantly enriched in WNT pathway (Figure 5a). Previous studies have demonstrated an interaction between DDX5 and β-catenin, further supporting its relevance in our investigation.^14,17,26–28^ Considering that VAMP8 promoted DDX5 degradation, we next examined the level of β-catenin in VAMP8 overexpression cells and control cells. Our results showed that β-catenin was indeed downregulated in VAMP8-overexpressing 143B and HOS cells (Figure 5b,c). Notably, β-catenin is capable of promoting EMT process in cancers.^29–31^

(a) GSEA analysis showed that high expression of DDX5 was enriched in WNT pathway. Normalized Enrichment Score (NES) = 2.290133, nominal p-value = 0 and FDR q-value = 2.1561095E-4.

(b-c) Western blotting analysis showed that VAMP8 overexpression could decrease β-catenin levels. Quantification of three experiments, Mean ± SEM, p(143B-VAMP8, 143B-Vector) = 0.036, p(HOS-VAMP8, HOS-Vector) = 0.017.

(d) GO enrichment analysis showed that “cadherin binding” was enriched (indicated by red line). Fold Enrichment = 11.325 and *p* = 3.239E-08.

(e-h) EMT markers were measured by western blotting in 143B and HOS cells. Quantification of three experiments, Mean ± SEM, E-cadherin: p(143B-VAMP8, 143B-Vector) = 0.015, p(HOS-VAMP8, HOS-Vector) = 0.009; N-cadherin: p(143B-VAMP8, 143B-Vector) = 0.032, p(HOS-VAMP8, HOS-Vector) = 0.01; Vimentin: p(143B-VAMP8, 143B-Vector) = 0.001, p(HOS-VAMP8, HOS-Vector) = 0.009.

To gain further insight into the molecular mechanisms involved, we performed Gene Ontology (GO) enrichment analysis using DAVID (https://david.ncifcrf.gov/tools.jsp), a functional annotation tool, on the 57 interaction proteins identified earlier. The analysis revealed a significant enrichment of the “cadherin binding” term (Figure 5d). Furthermore, we performed experiments to examine EMT marker proteins (E-cadherin, N-cadherin, Vimentin). In VAMP8-overexpressing cells, the epithelial marker (E-cadherin) was significantly elevated. On the contrary, mesenchymal markers (N-cadherin, Vimentin) were significantly downregulated (Figure 5e-h). Collectively, our data indicated VAMP8 promoted DDX5 degradation via the ubiquitin-proteasome system, inhibited WNT/β-catenin pathway and subsequently suppresses the EMT process, thereby suppressing OS metastasis.

### VAMP8 promotes autophagy flux

**Figure 6.**
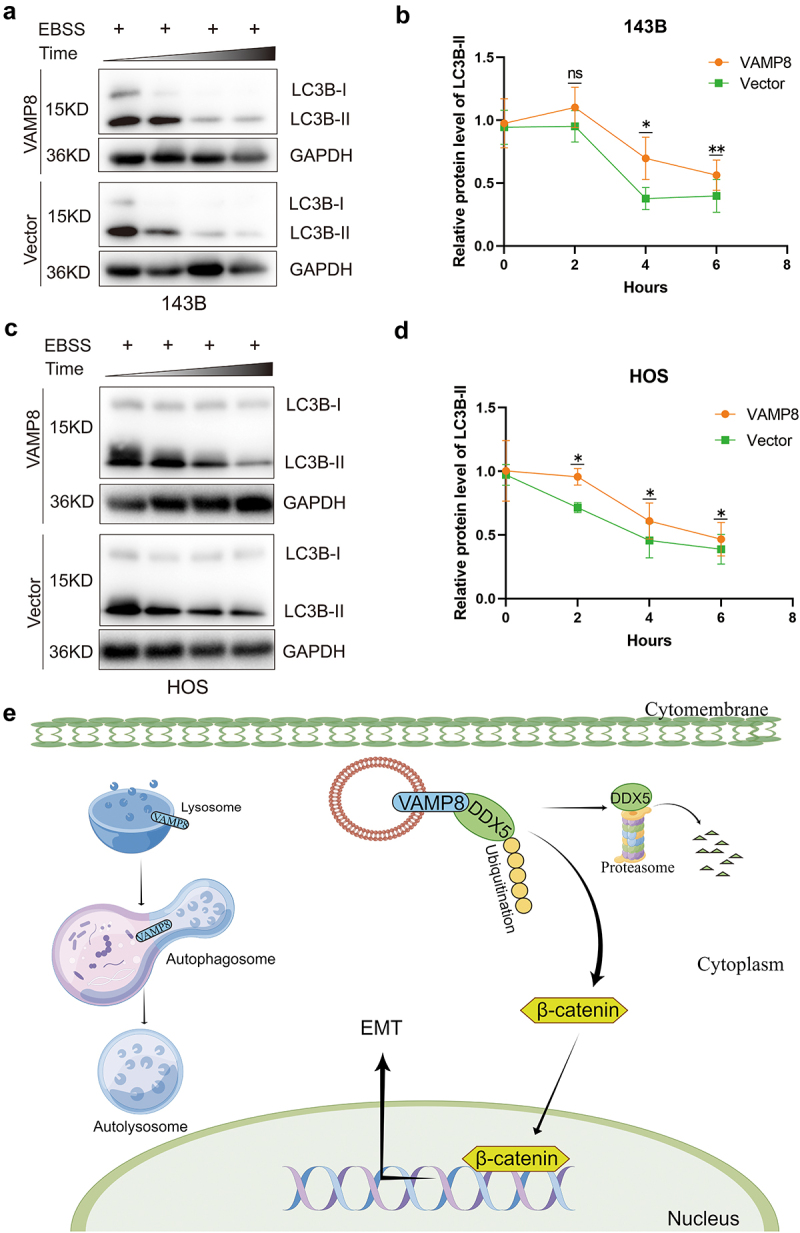
VAMP8 promotes the autophagy flux in OS cells.

Numerous studies have highlighted the inhibitory role of autophagy in tumor metastasis across various cancer types, including osteosarcoma (OS).^22,32^ VAMP8 was considered as a crucial protein to promote autophagosome-lysosome fusion and the autophagy flux.^33^ Accordingly, we examined whether the overexpression of VAMP8 promoted autophagy in OS cells. As anticipated, upon treatment with EBSS (Earle’s Balanced Salt Solution), we observed the protein level of LC3B is higher in VAMP8 stably expressing cells compared to the control cells (Figure 6a-d). These findings suggest that VAMP8 regulates autophagy flux, which may partly contribute to its role in suppressing metastasis in OS (Figure 6e).

(a-d) Western blotting analysis of LC3B levels in VAMP8 stably expressing cells and control cells treated with EBSS. After treatment for 4 or 6 h, *p* values were 0.033 and 0.002, respectively. In HOS cells, after treatment with EBSS for 0, 2, 4 or 6 h, *p* values were 0.022, 0.045, and 0.044, respectively. Quantification of three experiments, mean ± SEM, “ns”: not significant.

(e) Schematic diagram of VAMP8 functions in OS and the related mechanisms in this research. As demonstrated, VAMP8 promotes DDX5 degradation via the ubiquitin-proteasome system, inhibits β-catenin levels and further restrains EMT process. Meanwhile, autophagy is also dysregulated by VAMP8, which could be another potential mechanism.

## Discussion

This study has made several significant discoveries regarding the role of VAMP8 in osteosarcoma (OS) progression: (1) VAMP8 was downregulated in both OS tissues and OS cells. Overexpression of VAMP8 inhibited cancer cell migration and invasion, and low expression of VAMP8 promoted the progression of OS. (2) VAMP8 inhibited the OS metastasis by interacting with DDX5 and promoting its degradation via the ubiquitin-proteasome system. (3) VAMP8 overexpression inhibited the expression of β-catenin and the EMT process. Taken together, the above observations suggested that VAMP8/DDX5/β-catenin axis plays an essential role in OS metastasis and could be a molecular target for developing new OS treatments.

In this study, we initially analyzed the VAMP8 transcript in GEO database (GSE197158) and found the VAMP8 mRNA was downregulated in OS cells compared with hFOB1.19. However, Liu et al. reported that VAMP8 was upregulated in OS tissue compared with osteoblasts by analyzing GEO database (GSE14359, GSE16088 and GSE33383).^34^ It should be noted that their results have not been experimentally verified in OS cells and tissues yet. Conversely, our experiments demonstrated VAMP8 was reduced in both OS cells and surgery sectioned OS tissues. Moreover, we further showed that low expression of VAMP8 in OS patients was associated with poor overall survival rate. Therefore, we anticipated that VAMP8 was downregulated in OS and associated with the poor clinical prognosis. In addition, we found VAMP8 inhibited cancer metastasis in OS cells, which is in agreement with the study from lung cancer.^12^ We acknowledged that our current study was based on OS cells and patients’ tissues, and animal models were desirable in the future study to demonstrate the physiological role of VAMP8. Notwithstanding its limitation, this study does indeed indicate VAMP8 plays an important role in the OS progression.

Here we report, for the first time, the VAMP8 interacting partners in OS cells, which is important to decipher the molecular mechanisms underlying VAMP8 signaling in OS. Mass-spectrometry and immunoprecipitation experiments were performed to explore and confirm VAMP8 interacting partners in OS cells. We identified 57 related proteins possibly interacting with VAMP8. As the VAMP8 downstream targets could be regulated by interacting proteins, alterations in VAMP8 protein interacting partners may have direct clinical implications. We analyzed the clinical relevance of the top 10 proteins based on the closeness algorithms. Among them, the most clinically relevant protein was the RNA helicase DDX5. Interestingly, Chen et al. reported that the upregulation of DDX5 in OS is associated with poor survival.^18^ Previous studies have suggested vital roles of DDX5 in regulating the metastasis in types of cancer including OS.^18,35^ Accordingly, our results demonstrated that DDX5 is a target protein of VAMP8 mediating OS cell migration. VAMP8 overexpression results in a remarkably increased ubiquitination level of DDX5 and promotes its protein degradation.

We performed the GSEA analysis on the data from TARGET database and found that the high expression of DDX5 was enriched in WNT signaling pathway. β-catenin plays a central role in WNT signaling, and the WNT/β-catenin signaling pathway acts as a vital role in cell fate specification, migration and proliferation^36^. Dysregulated expression of β-catenin has been reported in many cancers including OS.^37,38^ A previous study reported that DDX5 can active β-catenin via promoting the nuclear translocation of β-catenin.^14^ Another study demonstrated DDX5 upregulated β-catenin and further prompted cancer metastasis.^17^ Consistently, our study indicated that the downregulation of DDX5 could reduce the protein level of β-catenin and consequently inhibit EMT process. However, these results including ours did not reveal the possibility of other potential targeting proteins involved in OS progression and illustrated the role of VAMP8 in vivo. Future investigations should aim to study the role of VAMP8 in vivo and explore other potential targeting proteins of VAMP8.

Autophagy plays complicated and double-sided roles in cancers. How autophagy influences cancers metastasis has been incompletely understood. Lysosome-localized VAMP8 was the key protein in SNARE complex-mediated autophagosome-lysosome fusion process.^39^ Consistent with the previous study,^39^ our result showed that overexpression of VAMP8 enhanced autophagy process. We consider that the low expression of VAMP8 may counteract OS metastasis via inhibiting autophagy. However, the exact molecular mechanisms remain to be explored in future studies.

In conclusion, our study uncovers a novel tumor-suppressive role of VAMP8 in OS and provides insights into the underlying molecular mechanisms. VAMP8 regulates OS metastasis by promoting the degradation of DDX5 through the ubiquitin-proteasome system, thereby downregulating β-catenin and inhibiting EMT. Furthermore, VAMP8 regulates autophagy flux, which may also contribute to OS progression. These findings highlight the potential of targeting VAMP8 and its associated pathways as a therapeutic approach for OS.

## Materials and methods

### Cell culture

Human OS cell line 143B, HOS, MG63, U2OS, normal human osteoblast cell line hFOB1.19 and human embryonic kidney cell 293T were acquired from American Type Culture Collection (ATCC, USA). hFOB1.19, HOS, MG63 and 293T were cultured in Dulbecco’s modified Eagle’s medium (DMED, Biological Industries, C3113–0500) supplemented with 10% FBS. 143B was cultured in Minimum Essential Medium (MEM, SparkJade, CL0001). U2OS was cultured in McCoy’s 5A Medium (Pricella, PM150710B). All of above cell lines were cultured in an incubator containing 5% CO_2_ at 37°C.

### Antibodies and chemicals

The following antibody was used in this study: VAMP8 (Proteintech, 15546–1-AP, 1:1000 for WB,1:500 for IHC), DDX5 (ZENBIO, R24094, 1:1000 for WB), DDX5 (Proteintech, 67025–1-Ig, 2 μg for IP), β-catenin (HUABIO, A6-F8, 1:2000 for WB), Vimentin (ZENBIO, R22775, 1:1000 for WB), E-cadherin (Proteintech, 20874–1-AP, 1:5000 for WB), N-cadherin (Proteintech, 22018–1-AP, 1:2000 for WB), ubiquitin (Proteintech, 10201–2-AP, 1:500 for WB), LC3B (Abmart, T55992, 1:1000 for WB), Flag (Proteintech, 66008–4-Ig, 1:5000 for WB), GAPDH (Affinity, AF7021, 1:10000 for WB).

The following chemicals were used in this study: Earle’s Balanced Salt Solution (Beyotime, C0213), Cycloheximide (MCE, HY-12320), Chloroquine (MCE, HY-17589A), MG132 (MCE, HY-13259).

### Clinical samples

Six pairs of clinical specimens were collected from the First Affiliated Hospital of Anhui Medical University. Acquisition of the donor OS tumor tissues and adjacent normal tissues and the following experimental procedures were approved by the Ethics Committee of Anhui Medical University (Approval number: 20200490; Date: 1st March 2020) and performed following the instructions from the Declaration of Helsinki. All the donors have provided written consent for the usage of the donor tissue in scientific research.

### Wound healing assay

143B and HOS were seeded into 12-well plates and cultured until a 100% confluence was reached. Then, the cells were scratched with a 200 μl pipette tip. The floating cells were washed twice with PBS, and cells were cultured in medium with 1% FBS. Photos were taken by a light microscope at 0, 12 or 24 h with ×40 magnification.

### Cell migration and invasion assay

The transwell assay was conducted to assess the migratory capability of OS cells using either a chamber (8 μm, LABSELECT) for the cell migration assay or a chamber precoated with Matrigel (ABW, 082724) for the cell invasion assay. 1 × 10^5^ 143B and HOS were seeded in each individual upper chamber of 24-well plates. Then, the 500 μL medium supplemented with 10% FBS was added to the lower chamber as a chemoattractant. After 12 h incubation for migration or 36 h incubation for invasion, cells migrating to the lower chamber were fixed in 4% paraformaldehyde for 30 min and then stained with crystal violet for 10 min. Finally, cells were photographed by a light microscope with ×40 magnification and three fields were randomly selected for cell counting.

### Plasmid construction, lentivirus production and infection

Plasmid construction was purchased from Tsingke Biotechnology (Nanjing, China). According to the manufacture’s instruction, lentiviral constructs of empty vector or Flag-VAMP8 were co-transfected with the virus packaging systems into 293T cells with Liposomal Transfection Reagent (YEASEN, 40802ES02). After 48 h of transfection, viral supernatant was harvested and centrifuged at 4000 rpm for 10 min. Then, HOS and 143B were infected using viral supernatant with Polybrene (YEASEN, 40804ES76). Stable cells were obtained in the presence of 4 µg/mL puromycin (Beyotime, ST551).

### Immunohistochemical (IHC), Hematoxylin and Eosin (H&E) staining assay

Paraffin-embedded tissue samples were cut into 3 μm thick sections for the next step of IHC and H&E assays. For IHC, the tissue sections were first dewaxed and boiled in sodium citrate buffer solution (Proteintech, PR30001) for 2 min to retrieve antigens. Then, tissue sections were closed with 3% H_2_O_2_ for 10 min to block endogenous peroxidase activity. Finally, sections were incubated with primary antibody at 4°C overnight. The next day, sections were washed three times with PBST and then sections were incubated with secondary HRP-conjugated antibody (MXB, KIT-5010) for 30 min. Then, sections were treated with DAB developing kit (Proteintech, PR30010). Finally, cell nuclei were stained with hematoxylin. For H&E, the sections were stained with hematoxylin for cell nuclei and eosin for cytoplasm.

Western blotting experiments were performed as described previously^40–43^. Briefly, cell proteins were extracted with RIPA lysate (Beyotime, P0013B) containing a cocktail of protease inhibitors (Beyotime, P1005) on ice for 30 min. The lysate was centrifuged at 12,000 rpm for 10 min. The supernatant was boiled at 100°C for 5 min and loaded into SDS-PAGE gel for electrophoresis. Then, proteins were transferred using polyvinylidene fluoride (PVDF) membranes and membranes were blocked with 5% nonfat milk for 90 min. Finally, the membranes were incubated with primary antibody overnight at 4°C. The next day, the membranes were washed three times with TBST and following incubated with secondary antibody for 60 min at room temperature. Eventually, the blots were detected by an ECL chemiluminescence system (Advansta, K-12043).

### Immunoprecipitation and mass-spectrometry

Cell proteins were extracted with lysis buffer containing protease inhibitor cocktail (Beyotime, P2181S) on ice for 10 min. The lysate was centrifuged at 12,000 rpm for 5 min. The supernatant reacted with anti-flag magnetic beads (Beyotime, P2181S) or control IgG magnetic beads (Beyotime, P2181S) overnight at 4°C on a rotator. The next day, the immunoprecipitated complexes were washed three times in lysis buffer and then eluted with 3X Flag peptide (Beyotime, P2181S) for 60 min at room temperature on a rotator. Then, the mixture was placed on a magnetic rack for 10 s. The supernatant was boiled at 100°C for 5 min and analyzed by Western blot.

The immunoprecipitated complexes were analyzed to identify the VAMP8 interaction partners with mass-spectrometry (Shanghai, Bioprofile Biotechnology).

### Cycloheximide (CHX) chase assays

For CHX assay, 5 × 10^5^ 143B or HOS cells were seeded into each well of 6-well plates. Next day, CHX was added into 6-well plates at a dose of 50 μg/ml. Cell proteins were extracted after treatment of 0, 3, 6 or 9 h, respectively. About 20 μg protein was used to examine DDX5 level by western blotting.

### Poly-ubiquitination assays

Poly-ubiquitination was measured in 143B and HOS cells. After treated with 10 μM MG132 for 9 h, cells were lysed with lysis buffer (Beyotime, P2181S). Proteins were immunoprecipitated to isolate ubiquitinated DDX5 with the anti-DDX5 antibody and then detected with the ubiquitin antibody by western blotting.

### Public database analysis

RNA transcriptome data were collected from TARGET, TCGA, CCLE and GEO database with series number GSE197158. We then analyzed the overall survival probability via using Kaplan–Meier curves and analyzed related molecular signaling pathway using GSEA. The pathway enrichment analysis used the c2.cp.kegg.v7.3.symbols.gmt gene sets of the official website. The enrichment analysis results of Gene Ontology (GO) biological process were obtained from DAVID (https://david.ncifcrf.gov/tools.jsp). Adjusted P-value <.05 was identified as significant and results were shown in the enrichment scatter plots. Cytoscape software (http://www.cytoscape.org) (version 3.9.0) was used to construct and visualize the PPI network according to the relationship between proteins of interest from the Search Tool for the Retrieval of Interacting Genes (STRING; http://string-db.org). Cytoscape’ plug-in molecular complex detection technology (MCODE) was used to examine the molecular complexes. Using the cytoHubba plug-in, the top 10 genes of the PPI network were defined as hub genes based on the closeness algorithms.^44^

### Statistical analysis

All experiments were repeated at least three times. Statistical analysis used GraphPad Prism 9.0.0 and data were expressed as the Mean ± SEM. *p* < .05, *p* < .01, *p* < .001 and *p* < .0001 are marked with *, **, *** and ****, respectively.

## Supplementary Material

Supplemental Material

Supplemental Material

## Data Availability

All the data generated during the current study are available from the corresponding author on reasonable request.
