# Supplementary figures and images for: VAMP8 suppresses the metastasis via DDX5/β-catenin signal pathway in osteosarcoma
Source: Cancer Biol Ther. 2023 Jul 5;24(1):2230641. doi: 10.1080/15384047.2023.2230641 (PMC10324439; doi:10.1080/15384047.2023.2230641)

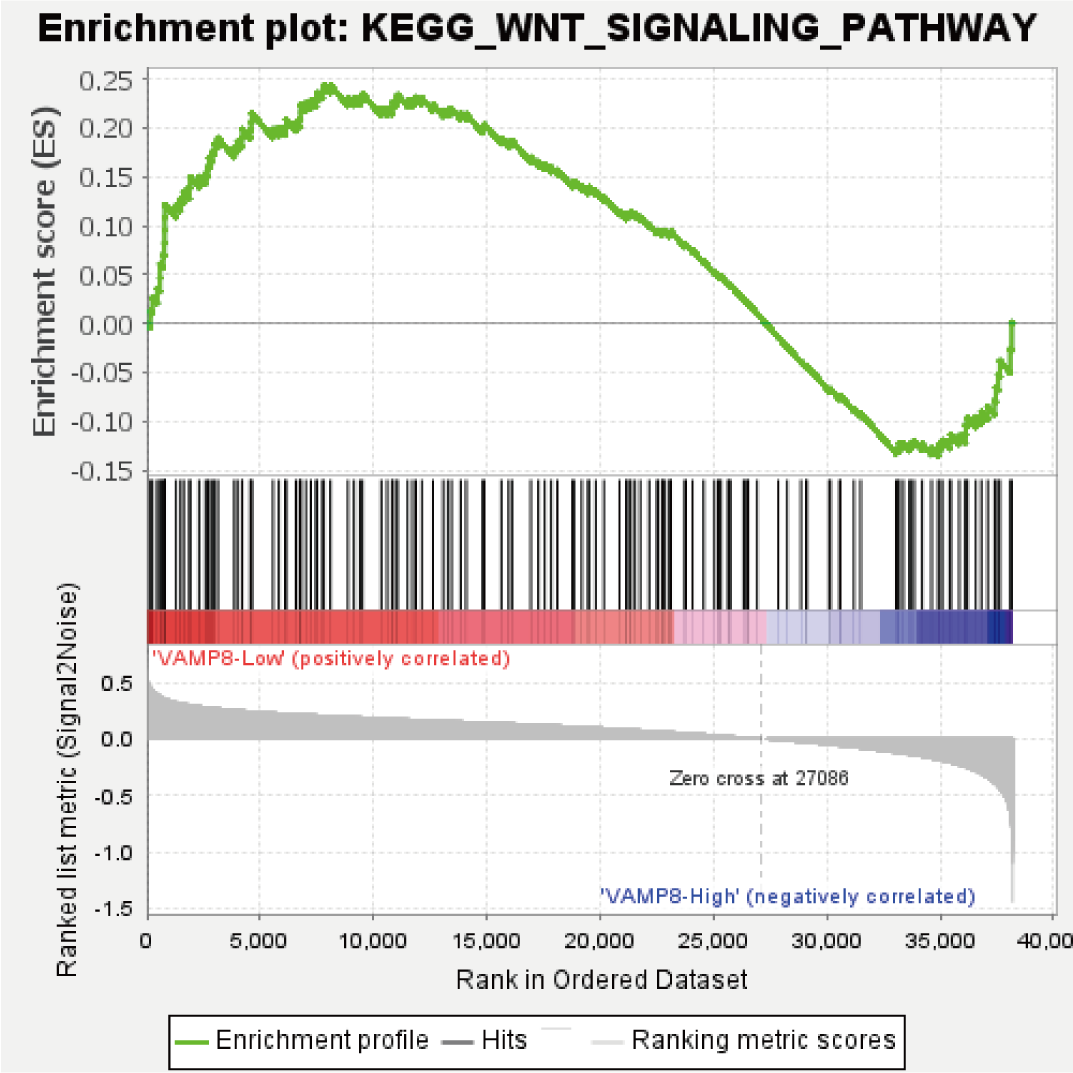

Supplement: Supplemental Material [file KCBT_A_2230641_SM2764.tif]

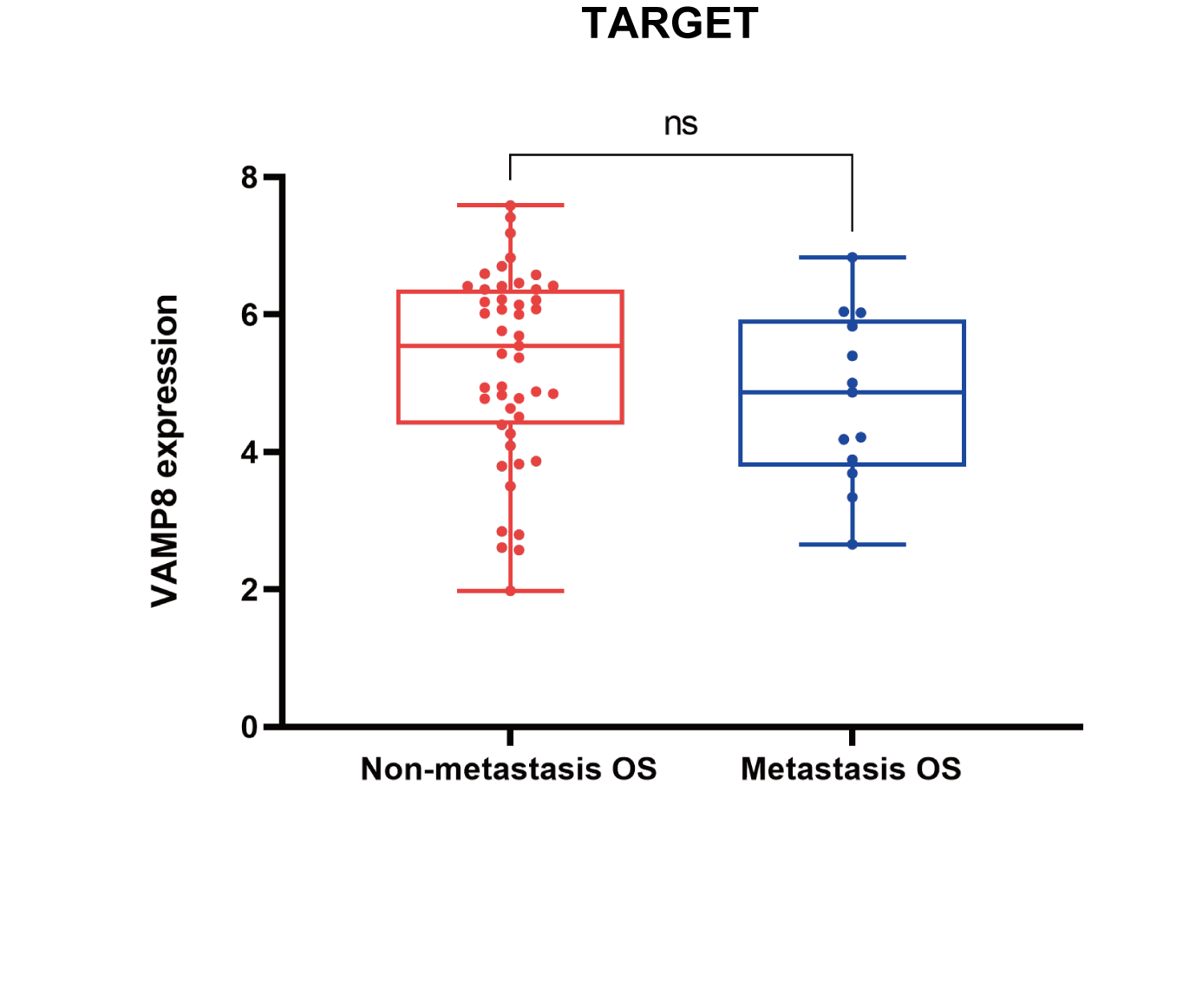

Supplement: Supplemental Material [file KCBT_A_2230641_SM2761.tif]
